# Special Issue “Clinical and Physiological Consequences of Hypoxia/Hypoxemia in Healthy Subjects and Patients”

**DOI:** 10.3390/jcm11133904

**Published:** 2022-07-05

**Authors:** Michelle Meyer, Aglaia Forrer, Martin Burtscher, Michael Furian

**Affiliations:** 1 Pulmonary Division, University Hospital Zurich, 8092 Zurich, Switzerland; micmeyer@student.ethz.ch (M.M.); aglaia.forrer@usz.ch (A.F.); 2 Department of Sport Science, University of Innsbruck, 6020 Innsbruck, Austria; martin.burtscher@uibk.ac.at

This editorial of the Special Issue “Clinical and Physiological Consequences of Hypoxia/Hypoxemia in Healthy Subjects and Patients” aims to draw more attention to the broad and diverse field of hypoxia research and serves as an invitation for research groups to share their most recent findings with the medical community. Hypoxia and hypoxemia are considered dangerous and harmful conditions that may affect a patient’s health and induce long-term damage to tissues and organs. In contrast, evidence is emerging that hypoxia and hypoxemia are not uniformly deleterious and can be used as therapeutic measures to improve outcomes.

**Hypoxia in the context of illness** is frequently associated with pulmonary diseases. It can be caused by reduced ventilation of the alveoli, impaired gas exchange, or paralysis of the respiratory musculature. A typical picture associated with hypoxemia is a distressed patient with chronic obstructive pulmonary disease (COPD) struggling to catch his breath. However, hypoxemia might also occur without clear symptoms, invisible to the observer or not noticeable for the patient. Many patients with chronic cardiorespiratory and other acute or chronic diseases are among millions of tourists exposed to hours and days of hypobaric hypoxia when travelling by airplane (normally pressurized to a maximum cabin altitude of about 2440 m [8000 ft]) [1] or when going to the mountains. In relation to the high number of air passengers and mountain tourists, only a few altitude-related adverse health effects occur, suggesting that a large proportion of patients with acute or chronic disease are able to tolerate a certain burden of hypoxia. In contrast, some patients respond highly sensitively to new-onset hypoxia, developing symptoms and requiring medical treatment even at moderate altitude [2]. It would be important to identify and advise such susceptible patients before they are exposed to hypoxia. Unfortunately, despite multiple efforts to establish a validated pre-flight or pre-ascent assessment tool, no evidence-based algorithm has been established yet [3]. Although short-term hypoxia may be tolerated even by chronically ill patients, Oldenburg et al. observed in stable heart failure patients with reduced ejection fraction that long-term hypoxemic burden (defined as the time of nocturnal oxygen saturation below 90%) was the most robust independent predictor of all-cause mortality, independent of sleep-disordered breathing [4]. Yet, another study showed no beneficial effects of long-term oxygen therapy in COPD patients with moderate hypoxemia (resting SpO_2_ of 89–93% or exercise-induced desaturations) to no use of supplemental oxygen. The two intervention arms did not differ significantly with regard to the rates of all-, COPD- and non-COPD-related deaths or first hospitalizations. In addition, quality of life, anxiety, depression, and other measures of functional status were unaffected by long-term oxygen therapy [5]. The two above-mentioned examples suggest that the effects of hypoxia and hypoxemia on clinical outcomes depend on the hypoxic pattern, severity, and duration, as well as the underlying disease. The example in COPD patients might direct the reader to the conclusion that mild to moderate hypoxia and hypoxemia might not be harmful and can be tolerated to a certain degree—a rather unexpected finding requiring further research.

**Hypoxia in the context of intervention** has a long history in sports medicine to improve physical performance in athletes by increasing their hemoglobin mass [6]. Moreover, altitude rehabilitation clinics are located at moderate altitude worldwide and are important therapeutic opportunities to recover from an exacerbation [7]. At low altitude, Burtscher et al. showed in a systematic literature review that hypoxic conditioning exerts several beneficial effects on the brain and could be an efficient intervention for numerous brain pathologies, and, moreover, that intermittent hypoxia has the potential to increase cerebral oxygenation in both men and women [8]. Further reported beneficial effects of hypoxia are myocardial protection, antihypertensive effects, prolonged anoxic survival of the brain by preserving its metabolism, neuroprotection in the retina, and finally, the possible improvement of regeneration following an organ insult [9]. In conclusion, and as previously emphasized in a review article by Navarrete-Opazo and Mitchell, the type of hypoxia exposure is crucial whether health effects are beneficial or detrimental [10]. These authors suggest that rather moderate hypoxia (FiO_2_: 9–16%) and low cycle numbers (not more than 15 episodes per day) are mostly associated with beneficial effects, while severe hypoxia (FiO_2_: 2–8%) and higher numbers of episodes (>48 per day) cause progressively greater pathology.

The above-mentioned examples and literature reviews highlight the complex role of hypoxia and hypoxemia (and attendant circumstances), and their important function in inducing beneficial or deleterious health effects. This Special Issue aims to further improve our understanding of preventing, controlling or inducing hypoxia and hypoxemia in healthy and ill persons.
